# Strengths Use for Tasks and Relationships in Organizations: Development and Validation of a Strengths Use Scale

**DOI:** 10.3389/fpsyg.2022.659046

**Published:** 2022-03-21

**Authors:** Shenyang Hai, In-Jo Park

**Affiliations:** Department of Psychology, Henan University, Kaifeng, China

**Keywords:** strengths use for tasks, strengths use for relationships, strengths use in organizations, character strengths, positive psychology

## Abstract

Individual character strengths have been increasingly valued, as they facilitate social functioning, well-being, and performance. However, little is known about how individuals use their strengths for important but distinct goals including task accomplishment and relationship maintenance in organizations. The purpose of this study is to develop and validate a Strengths Use Scale that can be used to measure the use of strengths for tasks and relationships in the workplace. For this purpose, we used the exploratory mixed-method design and conducted a series of studies. In Study 1, we conducted an exploratory factor analysis to ensure the construct validity of the Strengths Use Scale on a sample of 187 employees. We found that the scale comprises two dimensions: strengths use for tasks and strengths use for relationships. In Study 2a, we verified the two-factor structure of the Strengths Use Scale using the confirmatory factor analysis on a separate sample of 213 employees. The results of Study 2b demonstrated that the scale has good measurement invariance across gender and age groups, on the sample of 205 employees. Moreover, strengths use for tasks and strengths use for relationships positively correlated with well-being and work engagement and negatively correlated with turnover intention, supporting the criterion-related validity of the scale. In Study 3, a test–retest reliability analysis with a sample of 94 employees indicated that the scale has high reliability. Theoretical and practical implications of the findings are discussed.

## Introduction

Along with the emergence and development of positive psychology, individual character strengths have attracted increased attention from scholars and practitioners (van Woerkom et al., 2016a; Bakker et al., 2019). The positive psychology approach focuses on the investigation and application of the conditions and processes that may contribute to optimal functioning of individuals and organizations, which aims to complement the traditional deficit approach (Park et al., 2004; Gable and Haidt, 2005; Bakker and Derks, 2010). However, contemporary organizations still seem to devote greater efforts to individuals’ deficits or dysfunctions and invest in minimizing weaknesses (Bouskila-Yam and Kluger, 2011; Miglianico et al., 2020). Although such a deficit approach may help remediate individuals’ dysfunctions and lead to acceptable level performance, the focus on repairing weakness tends to be frustrating and less likely to promote individuals’ self-efficacy and positive affect (Hodges and Clifton, 2004; van Woerkom et al., 2016b). Researchers indicated that “minimizing weaknesses can prevent failure but cannot inspire excellence” (Miglianico et al., 2020, p. 738). In contrast, focusing on applying individual strengths has been suggested to be a more effective way to facilitate personal growth, development, and success (Linley et al., 2009; Bakker and van Woerkom, 2018; Ding et al., 2020). Therefore, it is of significance for the human resource management of contemporary organizations to shift from “repairing personal weaknesses” to “applying individual strengths,” thereby promoting organizational effectiveness.

Character strengths are the characteristics that allow an individual to perform well or at their personal best (Park et al., 2004; Wood et al., 2011). When individuals capitalize on their character strengths (e.g., humor, creativity, and social intelligence), they are more likely to be flourishing, feel energized, and deal with environmental challenges effectively (Lavy and Littman-Ovadia, 2017; Bakker et al., 2019). Previous studies have shown that the use of strengths leads to greater vitality, lower perceived stress, and sustainable well-being among a variety of groups including students, employees, and older adults (Wood et al., 2011; Douglass and Duffy, 2015; Dubreuil et al., 2016). In a work context, strengths use has been found to facilitate work engagement, organizational citizenship behavior, and job performance (Kong and Ho, 2016; van Woerkom et al., 2016c; Lavy and Littman-Ovadia, 2017; Yi-Feng Chen et al., 2021).

Despite a growing body of work on strengths use, we still know little about how individuals use their strengths for different goals in organizations and the outcomes of different types of strengths use. According to a literature (Bales, 1950; Beck et al., 2017), task goals and relational goals are the foundation of interactions in organizations. In other words, employees are not only needed to perform a set of prescribed tasks but also to interact with others (e.g., coworkers, supervisors, and customers) in the workplace (De Dreu and Weingart, 2003; Yang and Lau, 2015). Accordingly, employees may be required to devote efforts, adopt strategies, or use their strengths (e.g., creativity, gratitude, bravery, and social intelligence) to effectively deal with both task and relationship issues at work (Bakker and van Woerkom, 2018). For example, when confronted with the changing needs of customers, a service employee may use his or her strengths such as creativity to satisfy various customer requirements. When coworkers have relational conflicts at the office, a clerk can use strengths such as a sense of humor to relieve the interpersonal tension. Therefore, strengths use can be divided into strengths use for tasks and strengths use for relationships, just as leadership behaviors are classified into task-oriented and relations-oriented leader behavior in Ohio State Leadership Studies (Fleishman, 1953; Yukl, 1998). Researchers (Yukl, 1998; Fayyaz et al., 2014; Ceri-Booms et al., 2017) indicated that task and relational behaviors are distinct dimensions: supervisors’ task-oriented behavior is concerned about task completion and performance quality of followers, while relations-oriented behavior includes being considerate and supportive toward followers.

To further understand individuals’ strength use for tasks and strengths use for relationships, there is a need for validated scales to measure these phenomena. Although previous studies have developed several scales for measuring strengths use, the existing measurements (e.g., Govindji and Linley, 2007; Wood et al., 2011; van Woerkom et al., 2016b) treat strengths use as a single factor and are not able to capture different forms of strengths use in the workplace. As a result, we still have limited insights regarding how individuals use their strengths for important, but distinct, goals (e.g., task accomplishment and relationship maintenance) in organizations. To address the limitations of the existing strength use measurements, the current study aims to develop and validate a Strengths Use Scale in a work context, including the dimensions of strengths use for tasks and strengths use for relationships. For this purpose, we employed the exploratory mixed-method design and conducted several separate studies. In Study 1, we conducted an exploratory factor analysis (EFA) to examine the construct validity of the Strengths Use Scale on a sample of 187 employees. In Study 2a, we performed the confirmatory factor analysis (CFA) to ensure the factor structure on a sample of 213 employees. In Study 2b, we used multigroup CFA to test measurement invariance across gender and age and established the criterion-related validity of the measurement on a sample of 205 employees. In Study 3, a test–retest reliability analysis was conducted using data from 94 employees. The two-factor Strengths Use Scale may not only offer the opportunity for investigating and understanding the subtleties and complexities of employees’ strengths use at work but also provide implications for management to focus on the positive psychology approach and guide employees to apply their strengths for the achievement of different goals at work.

## Theoretical Background

### Character Strengths Theory and Strengths Use

Character strengths refer to positive characteristics of individuals that allow optimal functioning and performance (Park et al., 2004; Linley and Harrington, 2006). Examples of individuals’ character strengths are creativity, perseverance, kindness, gratitude, leadership, self-regulation, and humor, which are reflected in feelings, thoughts, and behaviors (Peterson and Seligman, 2004; Bakker et al., 2019). The character strengths theory suggests that everyone possesses certain character strengths, and these strengths may contribute to the social functioning and flourishing of individuals (Park et al., 2004; van Woerkom et al., 2016c; Lavy and Littman-Ovadia, 2017). For example, teachers’ individual strengths, such as social and emotional intelligence, are positively associated with their student’s academic performance; students with character strengths of perseverance and prudence are more likely to achieve better grades (Peterson and Park, 2006). Moreover, gratitude, honesty, self-regulation, zest, love, and hope are connected with a sense of calling; perseverance is strongly related to employees’ sense of meaning and performance at work (Smith, 2011; Littman-Ovadia and Lavy, 2016).

Although individuals’ character strengths tend to be stable, how character strengths are used is largely dependent on the context, interests, and personal values (Biswas-Diener et al., 2011; Bakker et al., 2019). For instance, when frontline service employees encounter angry customers, they may use their strengths such as emotional intelligence to calm down angry customers. Similarly, call center agents can use their social intelligence to establish good communication and interpersonal relationships with their clients. If individuals do not use certain strengths when needed, these strengths are less likely to help them achieve valued goals (Bakker and van Woerkom, 2018). It is significant and beneficial for individuals to possess several certain strengths; however, the use of strengths seems to be more critical in promoting valuable individual outcomes (e.g., work engagement, job satisfaction, and well-being) than the possession of strengths (Littman-Ovadia and Steger, 2010; Lavy and Littman-Ovadia, 2017).

We argue that the topic of strengths use deserves more research attention and deeper understanding for several reasons. First, organizational management tends to identify and correct individuals’ dysfunctional skills, attitudes, or behaviors through various training, coaching, and feedback (Linley et al., 2009; van Woerkom et al., 2016a). However, this deficit approach is demeaning and, therefore, less effective in promoting excellent performance (Kaiser and Overfield, 2011; van Woerkom and de Bruijn, 2016). Recently, researchers have suggested that the greatest opportunity for individual development and success lies in investing in their strengths rather than repairing their weaknesses (Bakker et al., 2019; Miglianico et al., 2020).

Second, recent studies have shown the beneficial effects of strengths use in the workplace (Dubreuil et al., 2016; Bakker et al., 2019). Individuals who actively use strengths are more likely to experience energy, authenticity, and a state of deep concentration, because they are doing what they naturally do best (Dubreuil et al., 2014). Moreover, organizations can increase employees’ productivity and reduce their absenteeism by supporting employees’ strengths use (Stander et al., 2014; van Woerkom et al., 2016a). Third, although character strengths are relatively stable, the ability to use strengths can be developed and improved through training programs such as strengths-based practices (Bakker and van Woerkom, 2018). If employees are trained to use their strengths to complete tasks and establish relationships with coworkers, they are more likely to be productive at work and maintain harmonious working relationships.

### Strengths Use for Tasks and Strengths Use for Relationships

Based on extant theory and research, the study of Govindji and Linley (2007) defined strengths use as the extent to which an individual uses his or her strengths in various settings. To measure strengths use, Govindji and Linley (2007) developed a Strengths Use Scale, which was validated in a sample of 214 college students. This is a single-factor scale including 14 measurement items. Based on Govindji and Linley’s (2007) Strengths Use Scale, Wood et al. (2011) developed and validated a general scale for strengths use that applies to adults. This scale has been demonstrated to be a reliable and valid measuring tool. However, the scale measures strengths use in a relatively broad context and does not specifically refer to the working context. For example, the item “I always try to use my strengths” from the scale by Wood et al. (2011) is difficult to clearly distinguish the use of strengths in the living area or the work area. Considering that there was no valid measurement to assess strengths use in organizational settings, van Woerkom et al. (2016b) developed and validated a new instrument that measures strengths use in the work context. This scale consists of 6 measurement items for measuring strengths use at work and has acceptable reliability and validity.

In the current study, we note that the existing single-dimension Strengths Use Scale may be not able to capture different forms of employees’ strengths use in the workplace. In organizational settings, employees not only need to achieve task-related goals but also to interact with others and deal with interpersonal relationships. The literature on organizational behavior has underscored the importance of two distinct goals—task accomplishment and relationship maintenance in organizations (van Woerkom and Meyers, 2015; Yang and Lau, 2015). Achieving high task performance is closely associated with pay rises and promotion, and establishing positive interpersonal relationships is helpful to improve employees’ belongingness, positive emotions, and needs satisfaction (e.g., Foulk et al., 2019; Alessandri et al., 2020; Park et al., 2021b). Therefore, employees’ strengths use may be divided into different types, depending on their goals. Moreover, according to self-determination theory (Ryan and Deci, 2000), individuals are innately motivated to fulfill their psychological needs such as needs for competence and relatedness, which are essential for human functioning (Kong and Ho, 2016). In order to satisfy needs for competence (i.e., the need to feel effective in the environment and capable of achieving desired outcomes), employees may actively make use of their strengths to facilitate excellent performance (van Woerkom et al., 2016b); to satisfy needs for relatedness (i.e., the need to feel connected to and cared for by others), they are able to use their strong points to build positive relationships with others at work. However, the instrument measuring strengths use for tasks and strengths use for relationships is not available in the literature, resulting in a limited understanding of how employees use their strengths to pursue task and relationship goals at work.

Based on the positive psychology literature and related theories (e.g., Park et al., 2004; Wood et al., 2011; van Woerkom et al., 2016b; Bakker and van Woerkom, 2018), this study sought to address the research gap by developing a Strengths Use Scale, which includes the dimensions of strengths use for tasks and strengths use for relationships. *Strengths use for tasks* refers to the use of personal strengths to accomplish work-related tasks. Examples of strengths use for tasks include using strengths to fulfill the requirements of the job and applying strengths to resolve difficulties of job-related tasks. *Strengths use for relationships* refers to the use of one’s strengths to create positive connections and establish relationships with others at work. Strengths use for relationships includes exerting one’s strong points to build positive interpersonal relationships with others at work and making most people at work feel comfortable by using one’s strengths. Since both forms of strengths use to capture the application of strengths at work, they fall well within van Woerkom et al.’s (2016b) conceptualization of strength use at work. Nonetheless, strengths use for tasks and strengths use relationships differ from each other: the former focuses on applying strengths to accomplish job-related tasks and goals, while the latter focuses on using strengths during interpersonal interactions and relationship building at work.

The literature on two predominant forms of leadership behavior – task-oriented behavior and relations-oriented behavior provides evidence that task and relations-oriented behaviors are conceptually different dimensions (e.g., Yukl, 1998; Kellett et al., 2006; Ceri-Booms et al., 2017). Leaders’ task-oriented behaviors are aimed at improving processes that facilitate task accomplishment, while relations-oriented behaviors concern maintaining or creating cooperative interpersonal relationships at work (Kellett et al., 2006; Fayyaz et al., 2014). Just as supervisors use task-oriented behavior to achieve task goals and exert relations-oriented behavior to maintain harmonious relationships (Yukl, 1998; Fayyaz et al., 2014), employees can also use their strengths to complete tasks and create positive connections with others at work. Previous studies (e.g., Settoon and Mossholder, 2002; Slemp and Vella-Brodrick, 2013) have indicated that employees may engage in different forms of behaviors to deal with task-related issues and build relationships with others.

The classification of strengths use for tasks and strengths use for relationships also draws from the framework of task and relational conflict (Jehn, 1995; Janssen et al., 1999; De Dreu and Weingart, 2003). According to Jehn (1995), employees in organizations would encounter two main issues – conflict based on the content of tasks and conflict based on workplace interpersonal relationships. Researchers (Jehn, 1995; Janssen et al., 1999) have differentiated task issues (i.e., task conflict) between relationship issues (i.e., relational conflict) in organizational settings. Accordingly, employees’ strengths use for tasks should also be distinguished from strengths use for relationships. Specifically, employees are likely to actively apply their strengths to engage in job-related tasks and solve task issues, but they may not necessarily use strengths for interpersonal relationship issues at work.

## Study 1: Scale Development and Construct Validity Analysis

Study 1 aimed to develop preliminary items for measuring strengths use, including dimensions of strengths use for tasks and strengths use for relationships, and to examine the construct validity of the developed measurement.

### Item Generation

Following the scale development guidelines suggested by scholars (Hinkin, 1995; DeVellis, 2003), we sought to develop a reliable and valid measure of strengths use. The guidelines include that items should reflect the purpose of the scale and the constructs, be clear and concise, and have appropriate reading difficulty levels (DeVellis, 2003). We generated new preliminary items based on the literature on strengths use in organizations and our derived definitions of the constructs. We also reviewed the relevant measures such as Peterson and Seligman’s (2004) Values in Action Inventory of Strengths, Govindji and Linley’s (2007) Strengths Use Scale, Wood et al.’s (2011) Strengths Use Scale, Keenan and Mostert’s (2013) Perceived Organizational Support for Strength Use Scale, van Woerkom and Meyers’s (2015) Strengths-Based Climate Scale, and van Woerkom et al.’s (2016b) the Strengths Use and Deficit Correction questionnaire. For instance, referring to the Govindji and Linley’s (2007) scale, we generated a question related to strengths use for tasks, “I apply my strengths to achieve the objectives of the job,” and a question related to strengths use for relationships, “I am able to make most people at work feel comfortable by using my strong points,” based on the question in the Govindji and Linley’s (2007) scale, “I achieve what I want by using my strengths.” Referring to the van Woerkom et al.’s (2016b) scale, we created a question related to strengths use for tasks, “I use my strengths to accomplish job-related tasks,” and a question related to strengths use for relationships, “I exert my strengths to build positive interpersonal relationships with others at work,” based on the question in the van Woerkom et al.’s (2016b) scale, “I use my strengths at work.”

Preliminary items were developed by two researchers and revised based on the expert comments of industrial and organizational psychologists. After the experts’ evaluations on the face validity of each item, the preliminary items were evaluated in terms of clarity and intelligibility of content. Three doctoral students in applied psychology were invited to read the content of the items and identify unclear or ambiguous items. Based on their feedback, the researchers further refined the items. Finally, the items received language editing from a professional language editor. These efforts resulted in an initial list of 18 items, consisting of nine items for measuring strengths use for tasks and nine items for measuring strengths use for relationships. According to the recommendation of Govindji and Linley (2007), the questionnaire was introduced with the following statement: *The following questions ask you about your strengths, that is, the things that you are able to do well or do best.* The questionnaires were prepared in English and then translated into Chinese following the back-translation procedure (Brislin, 1986) so as to ensure equivalency of meaning. The English version and the back-translated version reached a high agreement. Participants were asked to rate their agreement with each item on a 7-point Likert-type scale ranging from 1 (*strongly disagree*) to 7 (*strongly agree*).

### Method

#### Participants and Procedure

In this study, we collected data from 187 employees (Sample 1) who are working in various industries (e.g., manufacturing, education, and information technology) in Chinese cities. The participants were selected according to the following criteria: full-time employees with more than 6 months of working experience in the current organizations. The study sample consisted of 82 men (43.9%) and 105 women (56.1%). The average age of participants was 29.5 years (*SD* = 6.66), and the average organizational tenure was 6.02 years (*SD* = 5.77). In terms of educations levels, 14.4% had received a high school degree or below, 20.9% had a 2-year college degree, 50.8% had an undergraduate degree, and 13.9% held a graduate degree. Participants occupied different positions, including rank-and-file employees (78.6%), low-level managers (20.3%), and middle-level managers (1.1%).

In order to recruit participants, we randomly contacted 240 full-employees through personal contact and invited them to attend our study. They were introduced to the purpose of the research and the importance of providing honest responses. We also assured them that their responses would be kept confidential and used only for research purposes. One hundred and eighty-seven employees volunteered to participate in the study and completed an online survey through their smartphones. Participants were required to respond to all items to finish the survey, and thus there was no missing or incomplete data in the analysis. Our sample size was appropriate for factor analysis, as rules of thumb suggest that an ideal ratio of participants to scale items is 10:1 (Nunnally, 1978; Boateng et al., 2018).

#### Analysis of Data

In Study 1, we firstly conducted a parallel analysis using SPSS 20 (IBM SPSS Inc., Chicago, IL, United States) to determine the number of constructs to retain (O’connor, 2000). In a parallel analysis, 1,000 random data sets were generated with a 95% CI. Using data from Sample 1 (187 employees), EFA was then performed based on a principal axis factoring analysis with varimax rotation (Fabrigar et al., 1999). The minimum Kaiser–Meyer–Olkin (KMO) of 0.60 is considered as necessary for factor analysis, and the value above 0.7 is regarded as ideal (Cerny and Kaiser, 1977). Items with factor loadings that are below 0.35 are suggested to be eliminated since they explain the limited variance of the latent construct (Floyd and Widaman, 1995; Boateng et al., 2018). Moreover, factors with an Eigenvalue higher than 1 are suggested to be suitable for retaining (Hinkin, 1995; Owens et al., 2016).

### Results of Exploratory Factor Analysis

The results of the parallel analysis suggested retaining two factors. Following the recommendation of previous research (Floyd and Widaman, 1995), items with loadings on the first factor higher than 0.35 or with cross-loadings on the second factor lower than 0.4 were retained. Two items (i.e., “I do not use my strengths to complete my job-related tasks,” and “I do not apply my strengths to establish positive relationships with others at work.”) were eliminated because they had loadings lower than 0.35 on the first factor. A possible explanation for this result may be that reverse-worded items are highly likely to cause response errors and therefore impair construct validity (Swain et al., 2008; Park et al., 2019). The factor analysis was re-conducted on the remaining items, which results in a 16-item, two-factor solution (8 items for strengths use for tasks and 8 items for strengths use for relationships).

The minimum KMO was 0.93 in our analysis, indicating the factorability of the data set. The first factor was termed strengths use for tasks and explained 33.71% of the variance (Eigenvalue = 5.39). The second factor was labeled strengths use for relationships and explained 28.16% of the variance (Eigenvalue = 4.51). Together, the two factors accounted for 61.86% of the variance. Cronbach’s alphas for strengths use for tasks, strengths use for relationships, and for the overall items were 0.94, 0.91, and 0.94, respectively, demonstrating high reliability. To further confirm the internal consistency of the scale, we calculated the McDonald’s Omega values using JASP software. Omega has been suggested to be a more sensible and appropriate index of internal consistency compared with alpha (Dunn et al., 2014). In this study, the McDonald’s Omega values ranged from 0.82 to 0.94, which exceed the recommended cutoff of 0.70. These results provide initial support for the proposed two factors regarding strengths use. Scale items, item means, standard deviations, skewness, factor loadings, Cronbach’s alphas, and McDonald’s Omega are presented in Table 1. The Chinese version of the Strengths Use Scale is shown in the Appendix.

**TABLE 1:** Scale items statistics and factor loadings from exploratory factor analysis.

Items	*Mean*	*SD*	Skewness	Factor 1	Factor 2
**Strengths use for tasks**					
Q1. I use my strengths to accomplish job-related tasks.	5.79	1.00	−1.53	0.53	
Q5. I try to complete my tasks according to my competence.	5.80	0.93	−0.82	0.80	
Q6. I use my strong points to fulfill the requirements of my job.	5.69	0.97	−0.72	0.74	
Q11. I try to exert my strengths to make progress on job-related tasks.	5.80	0.96	−0.92	0.81	
Q12. I utilize my strengths to resolve the difficulties of job-related tasks.	5.80	0.84	−0.51	0.82	
Q13. I apply my strengths to achieve the objectives of the job.	5.78	0.96	−1.01	0.78	
Q15. In order to perform well on my job-related tasks, I play to my strengths.	5.76	0.87	−0.78	0.75	
Q16. I am used to completing job-related tasks in a manner that best suits my strong points.	5.66	0.91	−0.54	0.80	
**Strengths use for relationships**					
Q2. I exert my strengths to build positive interpersonal relationships with others at work.	5.80	0.90	−0.97		0.64
Q3. I establish positive relationships with others at work in a way I excel.	5.81	0.93	−1.00		0.65
Q4. I make the most of my strengths to resolve interpersonal conflict with others at work.	5.66	1.01	−0.98		0.79
Q7. I am able to make most people at work feel comfortable by using my strong points.	5.57	0.93	−0.66		0.62
Q8. In order to get on well with others at work, I capitalize on my strengths.	5.74	0.81	−0.28		0.74
Q9. I try to exert my strong points to gain interpersonal trust from others at work.	5.74	0.92	−0.70		0.74
Q10. In order to develop cooperative and trust-based relationships with others at work, I make use of my strong points.	5.65	0.89	−0.40		0.67
Q18. I use my talents to help others at work solve personal problems and cope with stress.	5.63	0.88	−0.29		0.65
Eigenvalues				5.39	4.51
Cumulative percentage of variance				33.71%	28.16%
Cronbach’s alpha (subscales)				0.94	0.91
McDonald’s Omega (subscales)				0.94	0.82
				CI = [0.92, 0.95]	CI = [0.78, 0.86]
KMO = 0.93, Bartlett χ^2^(df) = 2218.35 (120)***					

N = 187. Items with factor loadings less than 0.35 were not included in the table. KMO, Kaiser–Meyer–Olkin; df, degree of freedom; CI, confidence interval. ***p < 0.001.

## Study 2A: Confirmatory Factor Analysis and Factor Structure

The purpose of Study 2 was to investigate the factor structure of strengths use by conducting a set of CFAs. We expected that the two-factor model (including the factor of strengths use for tasks and the factor of strengths use for relationships) is best suitable for explaining strengths use.

### Method

#### Participants and Procedures

In this study, data were collected from service employees from various hotels in China. The targeted hotels were located in a city in the mid-eastern province of China. The participants were full-time employees with more than 6 months of working experience in the current hotels. They worked in different departments, including the front desk, the food and beverage department, the sales department, and the HR department. In total, data from 213 service employees (Sample 2a) working in 31 hotels were used in Study 2. The sample consisted of 84 men (39.4%) and 129 women (60.6%). The mean age of participants was 38.69 years (*SD* = 10.98), and the mean organizational tenure was 10.27 years (*SD* = 8.61). Their educational levels were high school and below (48.4%), 2-year college (39.9%), and undergraduate degree (11.7%). Regarding positions, 77.9% were rank-and-file employees, 21.1% were low-level managers, and 0.9% were middle-level managers.

In order to recruit participants, we randomly contacted 290 service employees from targeted hotels and invited them to attend our study. We explained the purpose of the research and highlighted the importance of providing honest responses. We also assured them that their participation was voluntary and confidential. In total, 213 employees took part in the study and completed the online survey through their smartphones. Strengths use was assessed using the measurement developed in Study 1. Based on the settings of the online survey system, participants were required to respond to all questions to complete the survey.

#### Analysis of Data

We conducted the CFA with the maximum likelihood estimation method *via* AMOS 17 (IBM SPSS Inc., Chicago, IL, United States) to examine the factor structure that best fit the data (Babakus et al., 1987). The fit indices include chi-square statistic, comparative fit index (CFI), Tucker-Lewis index (TLI), incremental fit index (IFI), standardized root mean squared residual (SRMR), and the root mean square error of approximation (RMSEA) were used to evaluate the model fit. Consistent with the recommendation of previous research (Hu and Bentler, 1998), the CFI value ≥ 0.9, the TLI value ≥ 0.9, the IFI value ≥ 0.9, the SRMR value ≤ 0.08, and the RMSEA value ≤ 0.08 indicate acceptable fit. In addition, Akaike’s information criterion (AIC) and Bayesian information criterion (BIC) were used to assess the relative fit of our proposed model and alternative models (Akaike, 1987). The model with a smaller AIC/BIC value is regarded as the better fitting model (Van de Schoot et al., 2012). Also, a difference in the AIC value between models (ΔAIC) greater than 2 suggests the significance of the model difference (Jöreskog and Sörbom, 1993).

### Results of Confirmatory Factor Analysis

The fit indices of the CFA models are displayed in Table 2. Based on the recommendations of Myers et al. (2014) and Zheng et al. (2015), we examined several models: the unidimensional model, first-order all-factor correlated model, hierarchical second-order factor model, and confirmatory bifactor model. The unidimensional model predicted only one dimension of strengths use. The first-order all-factor correlated model included two distinct but correlated dimensions of strengths use. The hierarchical second-order factor model predicted that the two lower-order strengths use dimensions account for the overall strengths use factor. The confirmatory bifactor model included a general factor (i.e., strengths use), group factors (i.e., strengths use for tasks and strengths use for relationships), and a loading matrix with a bifactor structure (i.e., each item loads on the general strengths use factor and may also load on a group factor). The Bifactor model is suggested as an important analytical approach to examine construct-relevant psychometric multidimensionality (Reise, 2012; Myers et al., 2014).

**TABLE 2:** Fit indices of the models from confirmatory factor analysis.

Models	χ^2^	df	CFI	TLI	IFI	SRMR	RMSEA	AIC	BIC
Model 1. Unidimensional	737.83	106	0.71	0.67	0.71	0.24	0.20	797.83	803.06
Model 2. first-order all-factor correlated	338.13	103	0.89	0.87	0.89	0.05	0.10	404.13	515.05
Model 3. Hierarchical second-order	309.44	102	0.91	0.89	0.91	0.05	0.10	377.44	491.72
Model 4. Confirmatory bifactor	269.85	88	0.92	0.89	0.92	0.05	0.10	365.85	527.19

*N = 213. CFI, comparative fit index; TLI, Tucker–Lewis index; IFI, incremental fit index; SRMR, standardized root mean squared residual; RMSEA, root mean square error of approximation; AIC, Akaike’s information criterion; BIC, Bayesian information criterion.*

**FIGURE 1:**
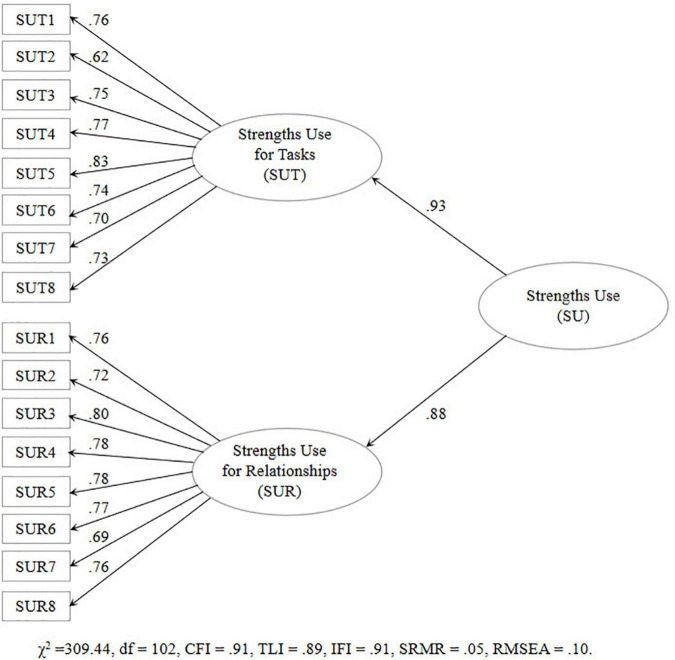
Hierarchical second-order factor model of strengths use.

As shown in Table 2, the hierarchical second-order factor model (χ^2^ = 309.44, df = 102; CFI = 0.91, TLI = 0.89, IFI = 0.91, SRMR = 0.05, RMSEA = 0.10) and the confirmatory bifactor model (χ^2^ = 269.85, df = 88; CFI = 0.92, TLI = 0.89, IFI = 0.92, SRMR = 0.05, RMSEA = 0.10) exhibited close fit and both of them showed acceptable model fit, supporting the dimensionality of the two-factor scale. Although the RMSEA values in the present study were slightly larger than the threshold value of 0.08, researchers (Hu and Bentler, 1998; Marsh et al., 2004; Zhang et al., 2019) suggested that the RMSEA value in the range from 0.08 to 0.1 indicates mediocre fit, and the value greater than 0.10 indicates a poor fitting model. The hierarchical second-order factor model was better than the alternative models, including the unidimensional model (χ^2^ = 737.83, df = 106; CFI = 0.71, TLI = 0.67, IFI = 0.71, SRMR = 0.24, RMSEA = 0.2) and the first-order all-factor correlated model (χ^2^ = 338.13, df = 103; CFI = 0.89, TLI = 0.87, IFI = 0.89, SRMR = 0.05, RMSEA = 0.1). Moreover, the AIC and BIC value of the hierarchical second-order factor model (AIC = 377.44; BIC = 491.72) was smaller than the alternatives (e.g., the unidimensional model and the first-order all-factor correlated model), and the differences were significant (ΔAIC > 2). The findings provided further evidence that strengths use is a two-factor structure (Zheng et al., 2015). The hierarchical second-order model is presented in Figure 1. The standardized factor loadings of measurement items were all high, ranging from 0.62 to 0.83.

Cronbach’s alpha was 0.94 for the overall strengths use. McDonald’s Omega for the overall strengths use was 0.94 (CI = [0.93, 0.95]). The reliability of strengths use dimensions was also good. Cronbach’s alphas for the dimensions of strengths use for tasks and strengths use for relationships were 0.9, and 0.91, respectively. McDonald’s Omega for the dimension of strengths use for tasks was 0.9 (CI = [0.88, 0.92]) and for the dimension of strengths use for relationships was 0.92 (CI = [0.9, 0.93]).

## Study 2B: Measurement Invariance and Criterion-Related Validity

Following the procedures of previous studies (van Woerkom et al., 2016b; Žvelc et al., 2020), the purposes of Study 2b were to (1) investigate measurement invariance of the Strengths Use Scale across gender and age and (2) establish the criterion-related validity of the measurement. To assess the criterion-related validity, this study focused on the correlations of strengths use for tasks and for relationships with employee well-being (i.e., cognitive and affective evaluations of the quality of an individual’s life; Diener, 1984), work engagement (i.e., a fulfilling and positive work-related state of mind characterized by vigor, dedication, and absorption; Schaufeli and Bakker, 2004), and turnover intention (i.e., the intention to change jobs voluntarily; Meyer and Allen, 1984). Prior studies have shown that strengths use is theoretically and empirically associated with well-being, work engagement, and turnover intention, since employees who use their strengths are more likely to experience positive states (e.g., feeling invigorated and competent) and perform effectively at work (e.g., van Woerkom et al., 2016b; Bakker et al., 2019; Yi-Feng Chen et al., 2021). Accordingly, we expected that strengths use for tasks and relationships are positively related to well-being and work engagement and are negatively related to turnover intention.

### Method

#### Participants and Procedure

In this study, data were from 205 employees (Sample 2b) working in different industries in Chinese cities. The study sample consisted of 80 men (39.0%) and 125 women (61.0%). The mean age of participants was 28.31 years (*SD* = 7.38), and the mean organizational tenure was 4.2 years (*SD* = 6.35). Regarding education levels, 17.6% had received a high school degree or below, 18.5% had a 2-year college degree, 46.3% had an undergraduate degree, and 17.6% held a graduate degree. In terms of positions, 74.1% were rank-and-file employees, 16.6% were low-level managers, 6.8% were middle-level managers, and 2.5 were high-level managers.

In total, we randomly invited 280 employees to participate in the study through personal contact. As in Study 2a, we explained the research purpose to them and assured them of data confidentiality and anonymity. In total, 205 employees volunteered to take part in the study and completed an online survey assessing demographic variables and main study variables.

#### Analysis of Data

We used multigroup CFA to test measurement invariance across gender and age *via* AMOS 17 (IBM SPSS Inc., Chicago, IL, United States). Measurement invariance reflects the extent to which the factor structure is generalizable across different groups (Vandenberg and Lance, 2000; Boateng et al., 2018). To determine measurement invariance, we compared several nested models step-by-step, including models examining configural invariance (the parameters are freely estimated in the groups while the factor structure is invariant across groups), metric invariance (the factor loadings of items are equivalent), and scalar invariance (item factor loadings, item intercepts, and means are invariant; Vandenberg and Lance, 2000; Zheng et al., 2015). We then calculated correlations *via* SPSS 20 (IBM SPSS Inc., Chicago, IL, United States) to investigate the criterion-related validity.

#### Measures

Following a back-translation procedure (Brislin, 1986), all the English items were translated into Chinese.

##### Strengths Use for Tasks and Strengths Use for Relationships

Strengths use for tasks and strengths use for relationships were assessed with 16 items of the newly developed measurement (see Table 1). Participants rated each item on a 7-point scale, ranging from 1 (*strongly disagree*) to 7 (*strongly agree*). Cronbach’s alphas for strengths use for tasks and strengths use for relationships were 0.95 and 0.94, respectively. McDonald’s Omega for strengths use for tasks was 0.95 (CI = [0.93, 0.97]) and for strengths use for relationships was 0.94 (CI = [0.92, 0.96]).

##### Well-Being

Well-being was measured with 6 items of the Life Well-being Scale developed by Zheng et al. (2015). An example item is “Most of the time, I do feel real happiness.” Participants responded to each item on a 7-point scale from 1 (*strongly disagree*) to 7 (*strongly agree*). Cronbach’s alpha was 0.93 in this study. McDonald’s Omega for this scale was 0.93 (CI = [0.92, 0.95]).

##### Work Engagement

Work engagement was assessed by using 6 items from Schaufeli et al. (2006) and adapted by Bakker and Xanthopoulou (2009). An example item is “At my work, I feel bursting with energy.” Participants rated each item on a 7-point scale, ranging from 1 (*strongly disagree*) to 7 (*strongly agree*). Cronbach’s alpha for this scale was 0.94. McDonald’s Omega was 0.94 (CI = [0.92, 0.95]).

##### Turnover Intention

The turnover intention was measured with 4 items from Babakus et al. (2009). An example item is “I often think about quitting.” Participants responded to each item on a 7-point scale from 1 (*strongly disagree*) to 7 (*strongly agree*). Cronbach’s alpha was 0.94 in this study. McDonald’s Omega for this scale was 0.94 (CI = [0.92, 0.96]).

### Results

The results of multigroup CFA (i.e., gender-group and age-group) are presented in Table 3. Based on the recommendation of Cheung and Rensvold (2002), the values of ΔCFI and ΔTLI between two nested models less than or equal to 0.01 indicate measurement invariance across groups. The ΔCFIs and ΔTLIs (ranging from −0.01 to 0) between two models in the gender-group were smaller than 0.01, suggesting that measurement invariance (i.e., configural, metric, and scalar invariances) existed in the Strengths Use Scale across the different genders. Moreover, the ΔCFIs and ΔTLIs in the age-group were also less than 0.01, which supports the measurement invariance of the factor structure across age. These findings suggest that the psychometric properties of the current measurement are generalizable across gender and age.

**TABLE 3:** Results of the measurement invariance testing across gender and age.

Models	χ^2^	df	CFI	TLI	IFI	SRMR	ΔCFI	ΔTLI
Model 1. Configural invariance^a^	530.28	206	0.90	0.88	0.91	0.05		
Model 2. Metric invariance	547.89	222	0.90	0.89	0.90	0.05	0.00^c^	−0.01^c^
Model 3. Scalar invariance	548.43	223	0.90	0.89	0.90	0.05	0.00^c^	−0.01^c^
Model 4. Configural invariance^b^	581.31	206	0.88	0.86	0.88	0.07		
Model 5. Metric invariance	596.84	222	0.88	0.87	0.88	0.07	0.00^c^	−0.01^c^
Model 6. Scalar invariance	598.93	223	0.88	0.87	0.88	0.08	0.00^c^	−0.01^c^

*CFI, comparative fit index; TLI, Tucker–Lewis index; IFI, incremental fit index; SRMR, standardized root mean squared residual. ^a^N of combined gender-group = 205, with N of females = 125 and N of males = 80. ^b^N of combined age-group = 205, with N of the participants aging < 27 = 111 and N of the participants aging ≥ 27 = 94. ^c^ΔCFI and ΔTLI less than the cutoff (>0.01) suggested by Cheung and Rensvold (2002).*

We then established the criterion-related validity of strengths use for tasks and for relationships by examining relationships with employee well-being, work engagement, and turnover. Table 4 presents the descriptive statistics and correlations for the studied variables. The results showed that strengths use for tasks was positively related to well-being (*r* = 0.39, *p* < 0.01) and work engagement (*r* = 0.43, *p* < 0.01) and was negatively associated with turnover intention (*r* = −0.26, *p* < 0.01). Strengths use for relationships was positively related to well-being (*r* = 0.51, *p* < 0.01) and work engagement (*r* = 0.53, *p* < 0.01) and was negatively associated with turnover intention (*r* = −0.26, *p* < 0.01). Although strengths use for tasks had a relatively high correlation with strengths use for relationships, the results of CFA indicated that strengths use for tasks and for relationships are positively related but can be distinguished. Overall, the findings supported the criterion-related validity of the developed measurement.

**TABLE 4:** Means, standard deviations, and correlations among study variables.

Variables	*M*	*SD*	SUT	SUR	WB	WE	TI
Strengths use for tasks	5.48	0.84	–				
Strengths use for relationships	5.23	0.83	0.62**	–			
Well-being	4.66	1.17	0.39**	0.51**	–		
Work engagement	4.64	1.20	0.43**	0.53**	0.66**	–	
Turnover intention	3.61	1.55	−0.26**	−0.26**	−0.37**	−0.46**	–

N = 205. SUT, strengths use for tasks; SUR, strengths use for relationships; WB, well-being; WE, work engagement; TI, turnover intention. **p < 0.01.

## Study 3: Test–Retest Reliability

The purpose of Study 3 was to confirm the stability of our measure by using the test–retest reliability method. Data were collected at both Time 1 and Time 2, which were 4 weeks apart. The time lag of 4 weeks is appropriate to explore the stability of the measurement, since a 4-week interval is long enough to allow the variability in individuals’ attitudes and behaviors but also is short enough to allow some stability in individuals’ lives (Daniels and Guppy, 1994; Ouweneel et al., 2011). Additionally, previous studies (e.g., Park et al., 2019, 2021a) have demonstrated the test–retest reliability of their measurements using a 4-week interval.

### Method

#### Participants and Procedure

In Study 3, we recruited participants using convenience sampling. From various industries, 113 employees took a web-based survey at Time 1. After a period of 4 weeks (Time 2), the participants were asked to fill out the same survey again. After matching data collected at the two different time points, we obtained 94 valid responses (Sample 3). Among the 94 participants, 28 were men (29.8%) and 66 were women (70.2%). The mean age of the participants was 27.02 (*SD* = 8.17) and the mean tenure was 4.01 years (*SD* = 4.54). Their educational levels were high school and below (33%), 2-year college (11.7%), undergraduate degree (50%), and graduate degree (5.3%).

#### Analysis of Data

We utilized SPSS 20 (IBM SPSS Inc., Chicago, IL, United States) to ensure the test–retest reliability of the Strengths Use Scale. The test–retest reliability is used to evaluate the degree to which the measurement is stable over time, i.e., how consistent the participants’ scores are across time (Boateng et al., 2018). The test–retest reliability of the two subscales was examined by using intraclass correlation coefficients after a 4-week interval. Higher intraclass correlation coefficients indicate higher test–retest reliability.

### Results

The results showed that Cronbach’s alphas for the Strengths Use Scale at Time 1 and Time 2 were 0.95 and 0.92, respectively (see Table 5). At Time 1, Cronbach’s alphas for strengths use for tasks and strengths use for relationships were 0.94 and 0.94, respectively; at Time 2, Cronbach’s alphas for strengths use for tasks and strengths use for relationships were 0.95 and 0.93, respectively. McDonald’s Omega ranged from 0.94 to 0.95 at Time 1 and ranged from 0.9 to 0.95 at Time 2. The results also showed that the intraclass correlation coefficient of the Strengths Use Scale was significant (*r* = 0.74, 95% CI = [0.57, 0.86]). Additionally, strengths use for tasks at Time 1 was highly correlated (*r* = 0.77, 95% CI = [0.52, 0.91]) with strengths use for tasks at Time 2; strengths use for relationships at Time 1 were also highly correlated (*r* = 0.73, 95% CI = [0.55, 0.85]) with strengths use for relationships at Time 2. Moreover, the results of the t-test showed no significant difference between the Time 1 measurement and the Time 2 measurement at the level of alpha = 0.05. These results demonstrated that the Strengths Use Scale has sufficient test–retest reliability.

**TABLE 5:** Reliability of the measurement.

	Time 1		Time 2	
	Cronbach’s alpha	McDonald’s Omega	Cronbach’s alpha	McDonald’s Omega
Strengths use for tasks	0.94	0.95, CI = [0.93, 0.96]	0.95	0.95, CI = [0.94, 0.97]
Strengths use for relationships	0.94	0.94, CI = [0.92, 0.96]	0.93	0.93, CI = [0.90, 0.95]
Overall strengths use	0.95	0.95, CI = [0.93, 0.96]	0.92	0.90, CI = [0.87, 0.93]

*N = 94. CI, confidence interval.*

## Discussion

The main purpose of this study was to develop and validate the two-dimension Strengths Use Scale, which can be used in psychology research to measure the extent to which individuals use their strengths for tasks and for relationships at work. In Study 1, we developed 18 preliminary items to measure strengths use and explored two factors – strengths use for tasks and strengths use for relationships by using EFA. The results confirmed the construct validity of the measure and revealed a 16-item, two-factor structure of strengths use measurement (8 items for measuring strengths use for tasks and 8 items for measuring strengths use for relationships).

In Study 2a, based on CFA, we confirmed the construct validity of this measure using the model fit indices, including CFI, TLI, IFI, SRMR, AIC, and BIC. Since the unidimensional model fitted the data poorly, the one-factor model was not able to adequately represent the underlying factor structure of strengths use. By demonstrating that both the hierarchical second-order factor model and the confirmatory bifactor model were better than the alternative models, the results further supported that strengths use is a second-order structure. Accordingly, the two dimensions (i.e., strengths use for tasks and strengths use for relationships) of the Strengths Use Scale are relatively distinctive and they reflect different aspects of strengths use. In Study 2b, the factor structure of the Strengths Use Scale demonstrated measurement invariance across gender and age. The results may indicate that employees from different gender groups and those from different age groups who perceive the items of the Strengths Use Scale in a similar way. We also supported the criterion-related validity of the scale by demonstrating that strengths use for tasks and strengths use for relationships were positively correlated with well-being and work engagement and were negatively correlated with turnover intention.

Finally, the results of Study 3 demonstrated that the Strengths Use Scale had sufficient test–retest reliability. The findings showed that the Strengths Use Scale, including the subscales of strengths use for tasks and strengths use for relationships, had high internal consistency (Cronbach’s alphas ranging from 0.92 to 0.95, McDonald’s Omega ranging from 0.9 to 0.95) and significant intraclass correlation coefficients. In line with Govindji and Linley’s (2007) scale (Cronbach’s alpha = 0.95), Wood et al.’s (2011) scale (Cronbach’s alphas ranging from 0.94 to 0.97), and van Woerkom et al.’s (2016b) measurement (Cronbach’s alpha = 0.92), our scale also showed good reliability.

### Theoretical and Practical Implications

This study contributes to the literature in several ways. First, theoretically, it extends the research on strengths use by developing a reliable and valid two-factor Strengths Use Scale. Although researchers have developed several scales for measuring strengths use (Govindji and Linley, 2007; Wood et al., 2011; van Woerkom et al., 2016b), the single factor of strengths use is not able to measure different forms of strengths use in a work context. We argue that employees may use their strengths for two important but distinct goals in organizations: task accomplishment and relationship maintenance. The rationale is similar to what the leaders exert task-oriented behavior to facilitate task progress and exert relations-oriented behavior to maintain positive interpersonal relationships (Fayyaz et al., 2014; Ceri-Booms et al., 2017). To address the limitations of exiting single-factor strengths use measurements, we propose two factors that can capture strengths use at work: strengths use for tasks and strengths use for relationships. Strengths use for tasks describes the use of individual strengths to complete work-related tasks, and strengths use for relationships describes the application of individual strengths to create connections and establish relationships with others at work (van Woerkom et al., 2016b; Bakker and van Woerkom, 2018). Our measure is distinct from general single-factor strengths use measures and can be used to expand the research on strengths use. For example, organizational behavior research can investigate the beneficial effects of strengths use for tasks and strengths use for relationships on employee task performance and contextual performance. It would also be worthwhile to investigate whether strengths use for tasks and strengths use for relationships lead to sustainable employee well-being.

Second, this study makes an important contribution to psychological research by integrating the positive psychology approach to organizational behaviors. Many organizations still invest great levels of efforts and resources in training employees to repair their dysfunctional skills, attitudes, or behaviors (Linley et al., 2009; van Woerkom et al., 2016a). However, positive psychologists have suggested that nurturing and applying strengths is more effective in facilitating well-being and engagement and may naturally promote excellent performance compared to repairing weakness (Peterson and Seligman, 2004; Bakker et al., 2019; Miglianico et al., 2020). By investigating how individuals apply their strengths for tasks and relationships in a work context, this study answered recent calls for more research on employees’ positive experiences such as strengths use (van Woerkom et al., 2016a; Bakker and van Woerkom, 2018). Although research on strengths use in organizational settings is still in its early stages, we believe that this study could promote the development of strengths use theory by providing a reliable and useful strengths use measurement for future empirical studies.

Third, practically, our measure may help organizations to find a more effective way to improve employee task and relationship performance and to ultimately facilitate organizational effectiveness. If organizations offer training that aims to help employees identify and apply their strengths to pursuing task and relationship goals, employees are more likely to achieve better task and relationship performance at work. This is because the use of individual strengths for tasks and relationships generates feelings of vitality, confidence, and a sense of fulfillment (van Woerkom et al., 2016a; Lavy and Littman-Ovadia, 2017; Bakker et al., 2019). Moreover, supervisors who capitalize on their employees’ strengths will be more effective than supervisors who focus on repairing employees’ weaknesses (Bakker et al., 2019). Accordingly, supervisors should realize the importance of employees’ strengths use and provide support with employees to apply their strengths for tasks and relationships at work. For example, supervisors could give employees space to perform work tasks in a way that they are good at. Supervisors may also encourage employees to create stronger connections with others (e.g., supervisors, coworkers, and customers) by applying their strengths.

### Limitations and Future Research Recommendations

This research has some limitations that need to be addressed by future studies. First, although the Strengths Use Scale was demonstrated to be a reliable and valid measure through EFA, CFA, and test–retest reliability, we lack the understanding of the predictive effects of strengths use. Whether strengths use for tasks leads to increased task performance and strengths use for relationships facilitates harmonious working relationships? To address these questions, future studies are encouraged to gain more insights into the consequences and possible mechanisms of strengths use for tasks and strengths use for relationships. Second, we developed and validated the Strengths Use Scale based on the samples from China, which may raise the question regarding whether this measurement is appropriate for other cultural populations. Since the Strengths Use Scale was developed based on general theories (e.g., character strengths theory and strengths use theory) and was not restricted to a particular cultural context, this scale is expected to be applicable for employees from different countries. Moreover, recent studies have applied strengths use theory to various cultural contexts (e.g., China and Western countries) and demonstrated that strengths use has good psychometric properties among different working populations (e.g., Lavy and Littman-Ovadia, 2017; Duan et al., 2018; Bakker et al., 2019; Lin and Ding, 2019; Ding et al., 2020). Nonetheless, in order to enhance the generalizability of our measurement, future studies should be conducted to investigate the cross-cultural applicability of this measurement. Third, participants in Study 3 were required to rate the measure two times over a 4-week period to confirm the test–retest reliability of the measure. Considering that 4 weeks is a relatively short time interval and may lead to a carryover effect due to memory or practice (Allen and Yen, 2001; Park et al., 2019), future studies should extend the time interval to strengthen the reliability of our scale.

## Conclusion

The important role of strengths use in promoting individuals’ performance and well-being has recently been acknowledged by researchers and practitioners. This study helps to extend the strengths use literature by introducing two novel and significant forms of strengths use: strengths use for tasks and strengths use for relationships. Employees can be encouraged not only to make use of their strengths to achieve excellent performance but also to build high-quality relationships in the workplace. Our findings from multiple studies provide support for the reliability and validation of the two-factor Strengths Use Scale. This newly developed measurement offers a useful and reliable tool for researchers and practitioners to refine our understanding of strengths use. We hope that the present study can promote further development of this important area of research so as to bring more beneficial and meaningful influences on people’s professional lives.

## Data Availability Statement

The raw data supporting the conclusions of this article will be made available by the authors, without undue reservation.

## Ethics Statement

Ethical review and approval was not required for the study on human participants in accordance with the local legislation and institutional requirements. The patients/participants provided their written informed consent to participate in this study.

## Author Contributions

SH and I-JP designed this research. SH analyzed data and wrote the first draft. I-JP provided supervision and revisions. Both authors approved the final version of the manuscript for submission.

## Conflict of Interest

The authors declare that the research was conducted in the absence of any commercial or financial relationships that could be construed as a potential conflict of interest.

## Publisher’s Note

All claims expressed in this article are solely those of the authors and do not necessarily represent those of their affiliated organizations, or those of the publisher, the editors and the reviewers. Any product that may be evaluated in this article, or claim that may be made by its manufacturer, is not guaranteed or endorsed by the publisher.

## Appendix: Chinese Version of the Strengths Use Scale

(优势使用量表中文版)

**任务优势使用**

1. 我利用自己的优势完成与工作相关的任务。
2. 我试图以自己擅长的方式完成任务。
3. 我会使用自己的强项来满足我的工作要求。
4. 我尽力发挥自己的优势，以取得工作任务上的进展。
5. 我发挥自己的强项，以解决工作任务上遇到的困难。
6. 我利用自己的优势来实现工作目标。
7. 为了在工作任务中表现出色，我尽力发挥自己的长处。
8. 我习惯以最适合我优势的方式完成与工作相关的任务。

**关系优势使用**

1. 我发挥自己的优势与工作中的其他人建立积极的人际关系。
2. 我以自己擅长的方式与他人建立积极的关系。
3. 在工作中，我充分利用自己的优势去解决与他人的人际冲突。
4. 我运用自己的优势，使大多数人在工作中感到舒适。
5. 为了与他人在工作中相处融洽，我充分发挥自己的长处。
6. 我充分发挥自己的长处，以赢得工作中他人的人际信任。
7. 为了在工作中与他人建立合作信任关系，我利用自己的优势。
8. 我用自己的优势帮助工作中的其他人解决个人问题或应对压力。
